# The Expression of *Millettia pinnata* Chalcone Isomerase in *Saccharomyces cerevisiae* Salt-Sensitive Mutants Enhances Salt-Tolerance

**DOI:** 10.3390/ijms14058775

**Published:** 2013-04-24

**Authors:** Hui Wang, Tangjin Hu, Jianzi Huang, Xiang Lu, Baiqu Huang, Yizhi Zheng

**Affiliations:** 1 Shenzhen Key Laboratory of Microbial Genetic Engineering, Shenzhen Key Laboratory of Marine Bioresource and Eco-environmental Science, College of Life Sciences, Shenzhen University, Nanhai Ave 3688, Shenzhen 518060, Guangdong, China; E-Mails: whuisz@szu.edu.cn (H.W.); anlaganlag@gmail.com (T.H.); biohjz@szu.edu.cn (J.H.); luxiang@genetics.ac.cn (X.L.); 2 Institute of Genetics and Cytology, Northeast Normal University, 5268 Renmin Street, Changchun 130024, Jilin, China

**Keywords:** *Millettia pinnata*, flavonoid, chalcone isomerase, salt-tolerance

## Abstract

The present study demonstrates a new *Millettia pinnata* chalcone isomerase (MpCHI) whose transcription level in leaf was confirmed to be enhanced after being treated by seawater or NaCl (500 mM) via transcriptome sequencing and Real-Time Quantitative Reverse Transcription PCR (QRT-PCR) analyses. Its full length cDNA (666 bp) was obtained by 3′-end and 5′-end Rapid Amplification of cDNA Ends (RACE). The analysis via NCBI BLAST indicates that both aminoacid sequence and nucleotide sequence of the MpCHI clone share high homology with other leguminous CHIs (73%–86%). Evolutionarily, the phylogenic analysis further revealed that the MpCHI is a close relative of leguminous CHIs. The MpCHI protein consists of 221 aminoacid (23.64 KDa), whose peptide length, amino acid residues of substrate-binding site and reactive site are very similar to other leguminous CHIs reported previously. Two pYES2-MpCHI transformed salt-sensitive *Saccharomyces cerevisiae* mutants (Δ*nha1* and Δ*nhx1*) showed improved salt-tolerance significantly compared to pYES2-vector transformed yeast mutants, suggesting the MpCHI or the flavonoid biosynthesis pathway could regulate the resistance to salt stress in *M. pinnata*.

## 1. Introduction

**Figure 1:**
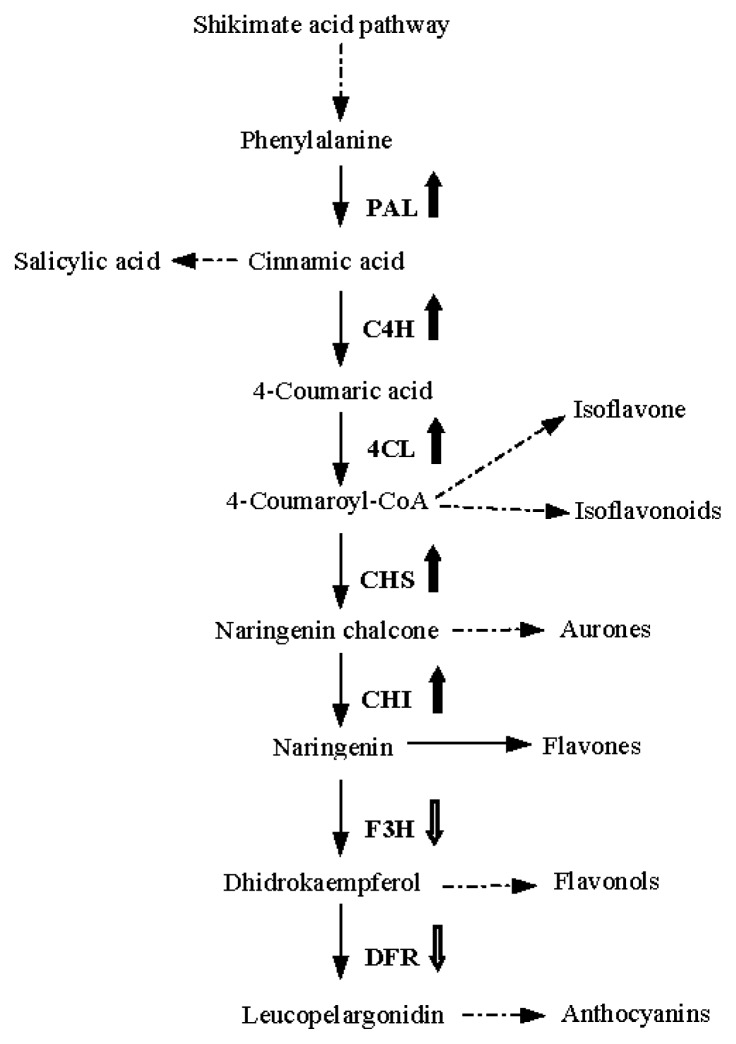
Outline of phenylpropanoid biosynthesis pathways in plants. Solid arrows indicate those single-step reactions; dashed arrows denote several steps. Enzymes are shown in bold. Thick bold arrows show those salt-inducible enzymes and blank thick ones demonstrate salt-repressed enzymes in *M. pinnata.* Abbreviations: PAL, phenylalanine ammonia-lyase; C4H, cinnamate 4-hydroxylase; 4CL, 4-coumarate:CoA ligase; CHS, chalcone synthase; CHI, chalcone isomerase; F3H, flavanone 3-hydroxylase; DFR, dihydroflavonol reductase.

*M. pinnata* (*Pongamia Pinnata*) belongs to the semi-mangrove plant which is an intervenient species between halophytes and glycophytes. It is the only mangrove plant in the leguminous family. Although *M. pinnata* does not have the salty gland that mangrove plants have, it can endure salt stress to a certain degree [1]. Therefore, the mechanism of *M. pinnata* salt-tolerance resembles glycophytes. It will play a vital role in improving salt-tolerance of crop plants by genetic modification, *esp.* leguminous plants, if the mechanism of *M. pinnata* salt-tolerance is clarified. Therefore, the analysis on the salt-tolerant transcriptome of *M. pinnata* has been performed utilizing Illumina sequencing in our lab. A series of genes with changed transcriptional level were observed [2], including those enzymes participating in plant secondary metabolism. Among them, the mRNA level of enzymes involved in flavonoid biosynthesis obtained the most remarkable change. Some of them showed enhanced transcription levels both in leaves and roots, such as phenylalanine ammonia-lyase (PAL), cinnamate 4-hydroxylase (C4H), 4-coumarate:CoA ligase (4CL), chalcone synthase (CHS) and chalcone isomerase (CHI), while the mRNA levels of flavanone 3-hydroxylase (F3H) and dihydroflavonol reductase (DFR) declined in roots after sea-water treatment (data not shown). It has been reported that the PAL enzyme is related to plants’ disease resistance [3,4]; CHS and CHI are the two key enzymes in plant flavonoid biosynthesis and were confirmed to be associated with UV protection [5–7]; while F3H and DFR, locating in the later steps next to CHS and CHI in phenylpropanoid biosynthesis pathway (Figure 1), are mainly responsible for the biosynthesis of anthocyanins which possess of photoprotection function [8]. The variations in their mRNA expression imply that CHS and CHI enzymes or their catalyzed flavonoids are closely associated with *M. pinnata* salt tolerance since both were improved in mRNA expression by salt stress.

Flavonoids have been reported to function in protecting plants against drought stress [9], toxic metal [6,10] and damage caused by UV [5–7,11]; however, their roles in plant salt-tolerance have been seldom reported. The responses of plant to salt stress are similar to those reactions to drought stress. Therefore, we would like to know the function of flavonoids in plant salt tolerance and the mechanism of the flavonoid biosynthesis in affecting or regulating plant salt tolerance. Moreover, we want to explore if there are common factors that could co-regulate the expression of enzymes in flavonoid biosynthesis pathway to increase or decrease when plants face salt stress. The CHI enzyme catalyzes the cyclization of chalcone into (2*S*)-naringenin which is the substrate both for flavones and anthocyanins. Once the biosynthesis of anthocyanins has been repressed, the metabolic flux will tend to the direction of flavone biosynthesis, indicating that flavones may be more vital to salt tolerance than anthocyanins in *M. pinnata*. Hence, the MpCHI was selected as a starting point to carry out this project.

In total, 12 putative *MpCHI*s have been annotated in the transcriptome sequencing assay in our lab. Only one of them was salt inducible, *esp.* in leaf, and chosen for the purpose of conducting this research. In this study, the QRT-PCR method was applied to further confirm that the *CHI* transcript level in *M. pinnata* leaves were dramatically improved 4 h after NaCl (500 mM) treatment. The *MpCHI* full length cDNA was obtained by RACE method and cloned into pYES2 yeast expression vector to generate plasmid pYES2-MpCHI. The pYES2-MpCHI transformed salt-sensitive *S. cerevisiae* deletion mutant strains: Δ*nha1* [12] and Δ*nhx1* [13] increased in tolerance to NaCl (1.2 M) in contrast to the pYES2-vector, without enzyme substrate fed into the culture medium, indicating one possibility that the MpCHI could regulate the response of *M. pinnata* to salt stress directly through changing its mRNA level or protein level.

## 2. Results and Discussion

### 2.1. The Transcription of MpCHI Is Up Regulated by 500 mM NaCl

**Figure 2:**
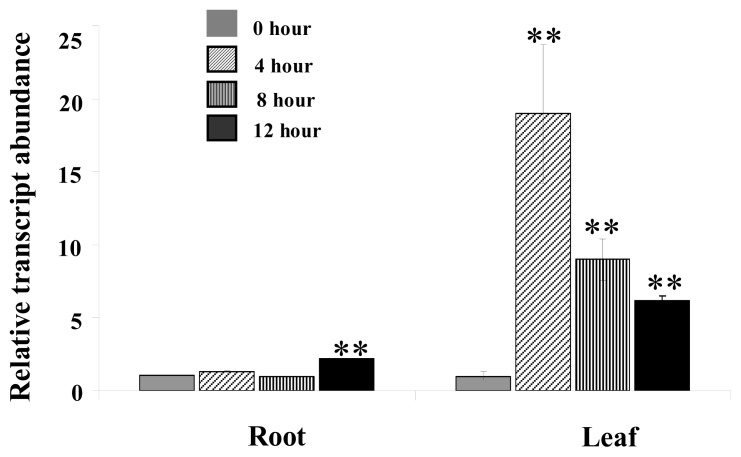
Quantitative real-time PCR analysis of *MpCHI* expression under salt stress. Leaf and root samples were collected at 0, 4, 8, and 12 h after 500 mM NaCl treatment. ** *p* < 0.01 by *U* test. Bars represent SD (*n* = 4).

To further confirm if the mRNA expression of *MpCHI* is salt inducible, the QRT-PCR assay was conducted to detect the relative transcription level of *MpCHI* in salt-treated *M. pinnata* roots and leaves (Figure 2). It was observed that the *MpCHI* transcription level was significantly enhanced (*p* < 0.01, *n* = 4) in leaves at 4 h (19 fold), 8 h (9 fold) and 12 h (6 fold) after salt-treatment, with the highest *MpCHI* mRNA amount at 4 h, verifying that the *MpCHI* cloned in this study is associated with salt-tolerance in *M. pinnata*. The reason why mRNA quantity decreased at 8 h and 12 h compared to 4 h might be that the *M. pinnata* plants had gradually adapted themselves to salt stress. This is consistent with the observation on *M. pinnata* leaf morphology when 20-d young plants were subjected to seawater treatment. The *M. pinnata* leaves wilted at 2 h and began to recover at 8 h (data not shown). In roots, slightly but significantly (*p* < 0.01, *n* = 4) incremental transcription quantity was also observed at 12 h (2 fold) after salt-treatment, indicating the expression of the *MpCHI* mainly occur in leaves. This is reasonable since the location of CHI in the flavonoid biosynthesis pathway is closed to the anthocyanin production which is mainly formed in plant overground tissues.

### 2.2. The MpCHI Shares High Homology with Leguminous CHIs

**Figure 3:**
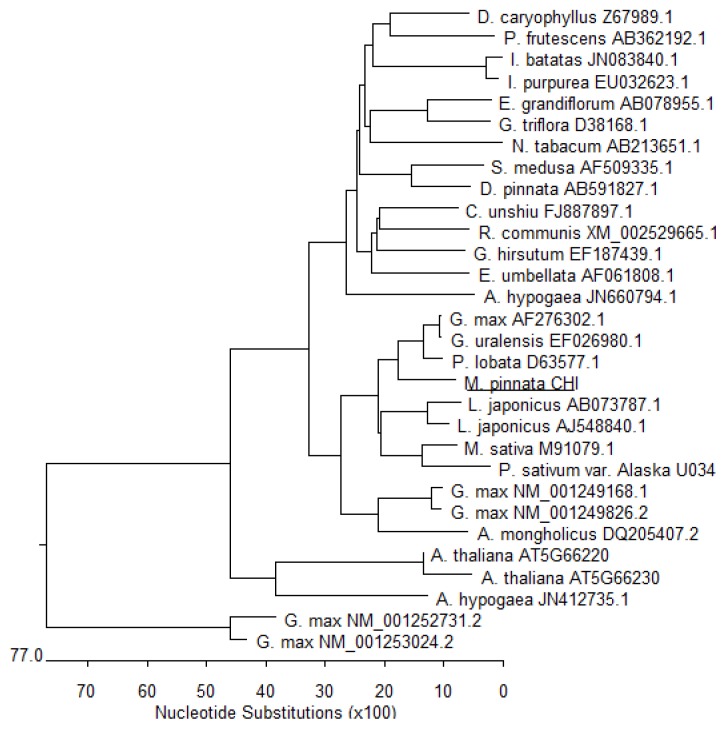
Phylogeny of plant chalcone isomerase (*CHI*) genes, based on 27 different plant *CHI* and *M. pinnata CHI* (*MpCHI*, underlined) nucleotide sequences cloned in this study, indicating *MpCHI* is a close relative of leguminous CHIs, with two soy bean *actin* cDNAs as control (Bottom *G. max* NM_001252731.2 and *G. max* NM_001253024.2). The accession numbers for the genes are displayed on the tree. Bottom scale shows nucleotide substitutions.

**Figure 4:**
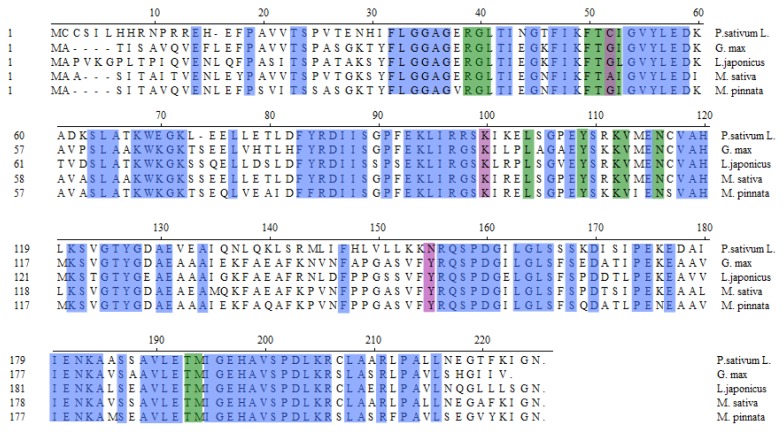
Peptide sequence alignment to compare MpCHI protein with four other leguminous CHIs. Residues of the (2*S*)-naringenin binding (green) and residues of the active site hydrogen bond network (purple) are highlighted according to previous report. Other conserved residues are marked in blue.

In order to verify if the *MpCHI* cDNA cloned here is correct, the putative *MpCHI* nucleotide sequence was analyzed using NCBI BLAST. The result indicated that both the MpCHI aminoacid sequence and nucleotide sequence (the open reading frame, ORF) (Figure S1) shares 85% homology with soybean CHI (GenBank: AF276302.1) peptide sequence and ORF nucleotide sequence. Sequence alignment was also carried out to compare the putative *MpCHI* ORF nucleotide sequence and other 27 plant CHI ORFs by phylogenic tree view (Figure 3), which demonstrated that the *MpCHI* is a close relative of leguminous plant *CHI*s. The peptide sequence alignment (Figure 4) showed that the aminoacid residues of substrate-binding site and reactive site in MpCHI enzyme are the same as other leguminous CHIs reported previously [14].

### 2.3. The pYES2-MpCHI Transformed Yeast Mutants Showed Improved Salt-Tolerance

**Figure 5:**
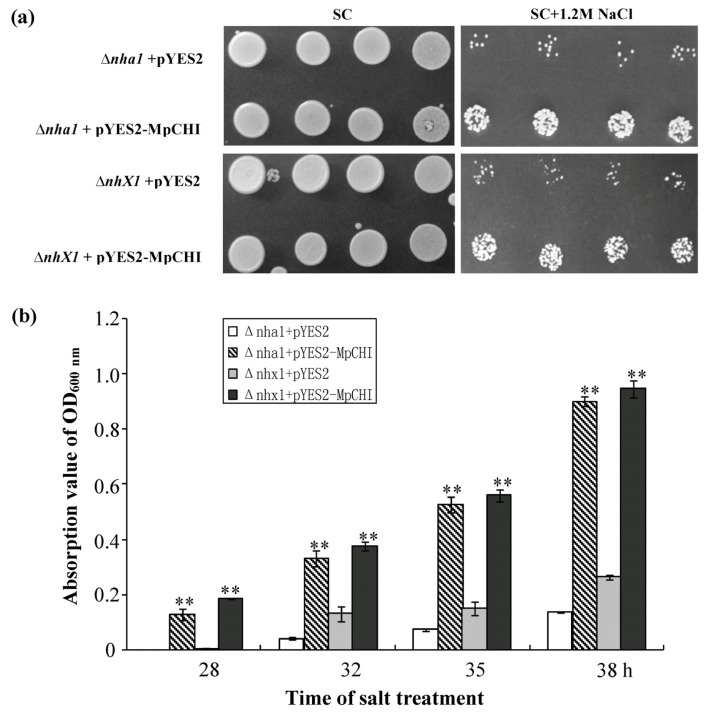
Enhanced salt tolerance in pYES2**-**MpCHI transformed yeast mutant. (**a**) Pictures were photographed 2 days after 1.2-M NaCl treatment, indicating the growth rate of pYES2-MpCHI and pYES2-vector transformed Δ*nha1* and Δ*nhx1* yeast mutants on SC agar plates with or without NaCl. Δ*nha1* + pYES2: empty vector transformed Δ*nha1* control; Δ*nha1* + pYES2-MpCHI: pYES2-MpCHI transformed Δ*nha1* yeast; Δ*nhx1* + pYES2: empty vector transformed Δ*nhx1* control; Δ*nhx1* + pYES2-MpCHI: pYES2-MpCHI transformed Δ*nhx1* yeast; (**b**) Absorption value of OD_600 nm_ to show the growth rate of pYES2**-**MpCHI and pYES2**-**vector transformed yeast in liquid medium containing 1.2-M NaCl. ** *p* < 0.01 by *U* test. Bars represent SD (*n* = 6).

Yeast has been successfully used as a model in exploring the function of plant flavonoid metabolic enzymes [15]. When soybean CHIs were expressed in yeast, proteins with CHI enzyme activity were obtained [15]. Hence, we utilized yeast to preliminarily probe the relationship between the MpCHI and salt response. Here, two *S. cerevisiae* salt-sensitive mutants (BY4742, Δ*nha1* and Δ*nhx1*) were transformed using plasmid pYES2-MpCHI to see the effect of MpCHI on their salt tolerance. Both of the pYES2-MpCHI transformed Δ*nha1* and Δ*nhx1 S. cerevisiae* strains grew faster (3.5–7.5 fold) than their corresponding pYES2-vector transformed control whether grown on agar plates (Figure 5a) or liquid medium (Figure 5b) containing 1.2 M NaCl.

We tried to transform wild-type (WT) yeast (BY4742) using pYES2-MpCHI, while the difference in salt resistance between the pYES2-vector transformed WT yeast control and the pYES2-MpCHI transformed WT yeast is not significant, because the WT yeast itself can resist NaCl-stress to a high concentration, even to 2.0-M NaCl. However, the yeast droplet could not be absorbed onto synthetic complete drop-out (SC) agar plates when the concentration of NaCl in SC medium increased to 2.5 M (data not shown). Hence, we tried to transform salt-sensitive *S. cerevisiae* deletion mutants, Δ*nha1* (BY4742; Mat α; his3Δ1; leu2Δ0; lys2Δ0; ura3Δ0; YLR138w::kanMX4), Δ*nhx1* (BY4742; Mat α; his3Δ1; leu2Δ0; met15Δ0; ura3Δ0; YDR456w:kanMX4) and Δ*hog1* (BY4742; Mat α; his3Δ1; leu2Δ0; lys2Δ0; ura3Δ0; YLR113w::kanMX4). Both NHA1 and NHX1 (NHA2) are involved in sodium and potassium efflux through the plasma membrane. They play significant roles in osmotolerance to heavy hypertonic stress [12,13]. We observed that the Δ*nha1* yeast decreased in tolerance to dehydration and osmotic stress [12,16] and Δ*nhx1* decreased in the tolerance to 1-M NaCl [13]. HOG1 is a mitogen-activated protein kinase and participates in osmoregulation by controlling the reallocation of RNA polymerase II in response to osmotic stress [17,18]. It was reported that the growth of Δ*hog1* was arrested in the presence of NaCl (0.4 M) [19]. The pYES2-MpCHI transformed Δ*hog1* did not show higher tolerance to either 400 mM or 800 mM NaCl than the pYES2-vector transformed Δ*hog1* control and could not even grow on 800 mM NaCl SC agar plates. Hence, the MpCHI could not reverse the effect aroused by HOG1 deletion and partially complement the deficiency in salt tolerance caused by NHA1 and NHX1 deletion (Figure S2), suggesting that MpCHI may has functional homology with NHA1 and NHX1 rather than HOG1. We also isolated a salt-inducible *M. pinnata CHS* (*MpCHS*) cDNA and introduced it into the same yeast mutants that *MpCHI* has transformed (data not shown). However, the *MpCHS* transformed yeast mutants only showed slightly faster growth than the empty vector transformants, suggesting that the *MpCHI* perhaps plays a more important role in salt tolerance in *M. pinnata* than the *MpCHS* does.

It has been verified that chalcone isomerase is unique in plants [13]. Therefore, there are no substrates for CHI in yeast. This assay insinuates that the MpCHI enzyme may participate in regulating the response of yeast to salt stress directly by three possibile manners. Firstly, it may play an osmotic regulation function in yeast transformants; secondly, it might help yeast transformants to reject the entering of salt ions; thirdly, the MpCHI may help to pump out the extra salt ions that have accessed to transgenic yeast. In *Arabidopsis*, three AtCHIs bearing fatty-acids binding function have been isolated [20]. Hence, the MpCHI could perhaps bind the membrane fatty acid of yeast cells and then influence the fluidity of cell membranes, finally either rejecting or pumping out the salt ions. To expound the exact mechanism about how MpCHI affects the salt tolerance in transgenic yeast or *M. pinnata*, further research is being performed in our lab.

## 3. Experimental Section

### 3.1. Salt-Treatment on *M. pinnata* Young Plants

*M. pinnata* seeds (Shenzhen Garden show park, China) were soaked in tap water at 28 °C in a growth cabinet until radicle appeared. These germinated seeds were then planted in soil for further 20-d growth and then submitted to salt (500-mM NaCl) and tap-water treatment. *M. pinnata* roots and leaves were sampled at different times (0, 2, 4, 6, 8 h) after treatment. Four plants were treated at each time point.

### 3.2. Real-Time Quantitative Reverse Transcription PCR (QRT-PCR)

Total RNAs were extracted from Salt-treated *M. pinnata* roots and leaves mentioned above using TRIzol reagent (Invitrogen, Carlsbad, CA, USA) following the instructions of manufacturer. RNA (2 μg) was used for the first-strand synthesis (20 μL reaction system) after treated by DNase following the instructions of the SuperScript™ First-Strand Synthesis System for RT-PCR (Invitrogen, Carlsbad, CA, USA). The RT product (1 μL) was applied as a PCR template to perform QRT-PCR in the ABI 7300 Real Time PCR System using the SYBR^®^ Premix Ex Taq™ (TaKaRa, Otsu, Japan) following the instructions of manufacturer with *M. pinnata Actin* (*MpActin*) as an internal control. Quadruple reactions were conducted. The same experiment was repeated 3 times and coincident results were obtained. The relative mRNA expression level was calculated and statistically analyzed using delta-delta-Ct method and *U*-test respectively, with non-treated samples as outer control. Primers CHI-IF (5′-CGGTGGCATCACTCGCCACC-3′) and CHI-IR (5′-TGCGGCGGCTTCAGCATCGCC-3′) were applied to amplify *MpCHI*; Primers ActinF (5′-AGAGCAGTTCTTCAGTTGAG-3′) and ActinR (5′-TCCTCCAATCCAGACACTAT-3′) were used for *MpActin* amplification.

### 3.3. *MpCHI* Full Length cDNA Clone

The 3′-end and 5′-end RACE were performed according to the instruction of FirstChoice^®^ RLM-RACE Kit (Roche). A nested PCR method was carried out to obtain the 3′-end and 5′-end of *MpCHI* cDNA. The gene-specific primer CHI-OF (5′-GATTACCTCTTCAGCCTCCGGC-3′) and the 3′-RACE outer primer provided by the manufacturer were used to do the first-round 3′-RACE PCR, whose product was then diluted 10 times and 1 μL of the diluted PCR product was applied as the template to do the second-round PCR by using the gene specific primer CHI-IF and the 3′-RACE outer primer. Both PCRs were finished under a temperature program of 94 °C, 30 s; 65 °C, 30 s and 72 °C, 90 s; running for 10 cycles; and then another temperature program of 94 °C, 30 s; 58 °C, 30 s and 72 °C, 90 s; running for 20 cycles. The PCR product from the second-round reaction was purified from argrose gel and then cloned into a T-vector (TaKaRa, Otsu, Japan) for DNA sequencing to obtain the 3′-end of *MpCHI* cDNA.

The Gene-specific primer CHI-OR (5′-CATGGTCTCCAACACTGCCTC-3′) and the 5′-RACE outer primer provided by manufacturer were used to do the first-round 5′-RACE PCR, whose product was then diluted 10 times and 1 μL was used as the template to do the second-round PCR by using the gene specific primer CHI-IR and the 5′-RACE outer primer. Both PCRs were finished under a temperature program of 94 °C, 30 s and 72 °C, 120 s; running for 5 cycles; and then 94 °C, 30 s; 65 °C, 30 s and 72 °C, 90 s; running for 10 cycles; after that, a final temperature program of 94 °C, 30 s; 58 °C, 30 s and 72 °C, 90 s; running for 15 cycles. The PCR product from the 2nd-round reaction was diluted 10 times and 1 μL of the diluted PCR product was applied as the template to do the third-round 5′-RACE PCR using gene specific primer CHI-IR2 (5′-GAAGTCGATGGCCTCTA CCAACTGTTC-3′) and 5′-RACE outer primer, under a temperature program of 94 °C, 30 s; 65 °C, 30 s and 72 °C, 90 s; running for 10 cycles; and then another temperature program of 94 °C, 30 s; 60 °C, 30 s and 72 °C, 90 s; running for 20 cycles. The PCR product from the third reaction was then cloned into the T-vector for DNA sequencing. The gene specific primers described above were designed according to the nucleotide sequence obtained from transcriptome sequencing (data not shown).

Primers CHI-F (5′-GCGTTTGCTGGCTTTGGTGAAAATAC-3′) and CHI-R (5′-GTTTTATTT CGTAACATAGTAGAAAGC-3′) designed according to the 3′-end and 5′-end *MpCHI* nucleotide sequence acquired from RACE assay were applied to amplify putative *MpCHI* cDNA. The PCR product (~900 bp) was cloned into the T-vector and was submitted to DNA sequencing. Subsequently, the sequence was analyzed by EditSeq of DNAstar software and searched a 666-bp ORF (Figure S1).

### 3.4. Phylogenic Analysis

In total, 27 plant *CHI*-nucleotide sequences (ORF) were found on the NCBI website. They are, *Arabidopsis thaliana CHI* (1638 bp, AT5G66230), *Arabidopsis thaliana CHI* 2 (525 bp, AT5G66220), *Arachis hypogaea* type I *CHI* (768 bp, GenBank: JN660794.1), *Arachis hypogaea CHI* (630 bp, GenBank: JN412735.1), *Astragalus mongholicus CHI* (660 bp, GenBank: DQ205407.2), *Citrus unshiu CHI* (669 bp, GenBank: FJ887897.1), *Dianthus caryophyllus CHI* (666 bp, GenBank: Z67989.1), *Dahlia pinnata CHI* (675 bp, GenBank: AB591827.1), *Elaeagnus umbellata CHI* (771 bp, GenBank: AF061808.1), *Eustoma grandiflorum CHI* (654 bp, GenBank: AB078955.1), *Gentiana triflora CHI* (666 bp, GenBank: D38168.1), *Glycine max* chalcone *CHI* (657 bp, GenBank: AF276302.1), *Glycine max CHI1B1* (681 bp, NCBI Reference Sequence: NM_001249826.2), *Glycine max CHI1B2* (681 bp, NCBI Reference Sequence: NM_001249168.1), *Glycyrrhiza uralensis CHI* (657 bp, GenBank: EF026980.1), *Gossypium hirsutum* cultivar *CHI* (684 bp, GenBank: EF187439.1), *Ipomoea batatas CHI* (732 bp, GenBank: JN083840.1), *Ipomoea purpurea CHI* (726 bp, GenBank: EU032623.1), *Lotus japonicus CHI* (636 bp, GenBank: AJ548840.1), *Lotus japonicus CHI* (678 bp, GenBank: AB073787.1), *Medicago sativa CHI-1* (669 bp, GenBank: M91079.1), *Nicotiana tabacum CHI1* (735 bp, GenBank: AB213651.1), *Perilla frutescens var. crispa CHI-1* (645 bp, GenBank: AB362192.1), *Pisum sativum var. Alaska CHI* (672 bp, GenBank: U03433.2), *Pueraria lobata CHI* (675 bp, GenBank: D63577.1), *Ricinus communis* putative *CHI* (531 bp, NCBI Reference Sequence: XM_002529665.1) and *Saussurea medusa CHI* (699 bp, GenBank: AF509335.1). Sequence alignment was carried out using MegAlign of DNAstar software by clustal V method, with two soy bean *actin* cDNAs as control (NM_001252731.2 and NM_001253024.2). Phylogenic tree was obtained by Phylogenic Tree View of MegAlign (Figure 3). The aminoacid sequences of MpCHI and four other leguminous CHIs were also committed to sequence alignment by clustal V method (Figure 4).

### 3.5. Plasmid pYES2-MpCHI Construction and Yeast Transformation

Primers WH01 (5′-ATGGATCCATGGCATCCATAACCGCAGTCC-3′) with *Bam*HI enzyme site underlined and WH02 (5′-ACGAATTCATCTCAAAAAGCTCAGTTGCC-3′) with *Eco*RI enzyme site underlined were used to amplify the full length *MpCHI* cDNA which was then cloned into the T-vector to generate plasmid pT-MpCHI. The MpCHI full length cDNA was excised from the plasmid pT-MpCHI (*Bam*HI*/Eco*RI) and cloned into *Bam*HI and *Eco*RI sites in pYES2/CT (Invitrogen, Carlsbad, CA, USA) yeast expression vector to form pYES2-MpCHI plasmid which is controlled by a GAL1 promoter [21,22]. Subsequently, the plasmid pYES2-MpCHI was mobilized into two salt-sensitive yeast deletion mutants (Δ*nha1* and Δ*nhx1*) which were purchased from EUROpean Saccharomyces Cerevisiae ARchive for Functional Analysis (EUROSCARF) using LiAc/SS carrier DNA/PEG yeast transformation method [23]. The plasmid pYES2 was also introduced into Δ*nha1* and Δ*nhx1* to generate two vector controls. The presence and expression of *MpCHI* were confirmed by PCR and RT-PCR (Figure S3) respectively using primer WH01 and WH02, with yeast *actin* (YFL039C) as control. Yeast RNAs were extracted using Yeast RNAiso Kit (TaKaRa, Otsu, Japan) Primers used for amplifying yeast *actin* are yACT1F (5′-CTACAACGAATTGAGAGTTGCC-3′) and yACT1R (5′-AACCAGCGTAAATTGGAACGAC-3′).

### 3.6. Salt-Tolerance Assay on the pYES2-MpCHI Transformed Δ*nha1* and Δ*nhx1* Yeast Mutants

The pYES2-MpCHI and pYES2 transformed Δ*nha1* and Δ*nhx1 S. cerevisiae* were firstly cultured in tubes containing 5 mL of SC liquid selection medium without uracil for 30 h at 30 °C, shaking at a speed of 250 rpm/min. One milliliter of each of the above yeast cultures was then transferred into 150-mL flasks with 50 mL of SC liquid medium for further 6 h incubation. The absorption value of OD_600 nm_ was then measured. Each yeast culture was diluted to OD_600 nm_ = 0.01 and 5 μL of the diluted culture was dripped onto the SC agar plates (Figure 5a) with or without NaCl (1.2 M) and liquid medium (Figure 5b) containing NaCl (1.2 M) to compare the growth rate, with galactose (2%) as a carbon source and inducing factor in gene expression. Agar plates were photographed 2 days after the salt-treatment assay and yeast cultured in liquid medium were measured the absorption value of OD_600 nm_ at 28, 32, 35 and 38 h after treatment.

## 4. Conclusions

In the present study, we isolated a new 666-bp *MpCHI* cDNA clone which has not been previously identified. Both its nucleotide and peptide sequence share 85% homology with soybean. The MpCHI protein possessed the substrate binding and reactive site as other leguminous CHIs have, suggesting the accuracy of *MpCHI* cDNA obtained in this study. Salt-sensitive yeast transformed by the plasmid pYEST2-MpCHI increased in salt tolerance, indicating the possibility that the MpCHI may be involved in *M. pinnata* salt tolerance in a direct manner. Future works should be carried out to introduce the *MpCHI* into plants to observe its effect on plant salt tolerance. The co-regulation manner among salt-inducible or salt-repressed enzymes in *M. pinnata* flavonoid biosynthesis is also an interesting project that we will perform in the future.

## Supplementary Information
