# Supplementary material for: The Expression of Millettia pinnata Chalcone Isomerase in Saccharomyces cerevisiae Salt-Sensitive Mutants Enhances Salt-Tolerance
Source: Int J Mol Sci. 2013 Apr 24;14(5):8775–86. doi: 10.3390/ijms14058775 (PMC3676755; doi:10.3390/ijms14058775)
Supplement: Supplementary file 1 [file ijms-14-08775-s001.pdf]

# Supplementary Information

**Figure S1.** (**a**) The nucleotide sequence of 666-bp open reading frame encoding MpCHI; (**b**) Agrose (1%) gel picture shows the 666-bp *MpCHI* band obtained by PCR. Lane 1, *MpCHI*; Lane 2, DNA molecular standard marker.

**(a)**

**ATGGCATCCATAACCGCAGTCCAGGTGGAGAACCTTGAGTTCCCATCGGTGATTACCTCTTCAGCCTCCGGCAAAA**

**CCTATTTCCTCGGCGGCGCAGGGGTGAGGGGGTTGACGATTGAGGGGAACTTCATAAAGTTCACAGGCATAGGAG**

**TATACTTGGAAGATAAAGCGGTGGCATCACTCGCCACCAAGTGGAAGGGTAAGACTTCAGAACAGTTGGTAGAGG**

**CCATCGACTTCTTCAGAGATATCATTTCAGGCCCATTTGAAAAGCTAATTCGAGGGTCCAAAATCAGAGAGTTGAG**

**TGGGCCGGAGTATTCAAAGAAGGTGATCGAAAATTCCGTGGCCCACATGAAGTCAGTTGGGACTTACGGCGATGC**

**TGAAGCCGCCGCAATTGAAAAATTTGCCCAAGCCTTCAAGCCTGTCAACTTTCCCCCGGGTGCCTCTGTTTTCTAC**

**AGGCAATCACCCGATGGAATCTTGGGGCTTAGCTTCTCTCAAGACGCAACATTGCCAGAAAATGAGGCAGCAGTTA**

**TAGAGAACAAAGCTATGTCAGAGGCAGTGTTGGAGACCATGATTGGGGAACACGCTGTTTCCCCTGATTTGAAAC**

**GCAGTTTGGCTTCAAGGTTCCCTGCCGTATTGAGCGAGGGCGTTTACAAGATTGGCAACTGA**

**(b)**

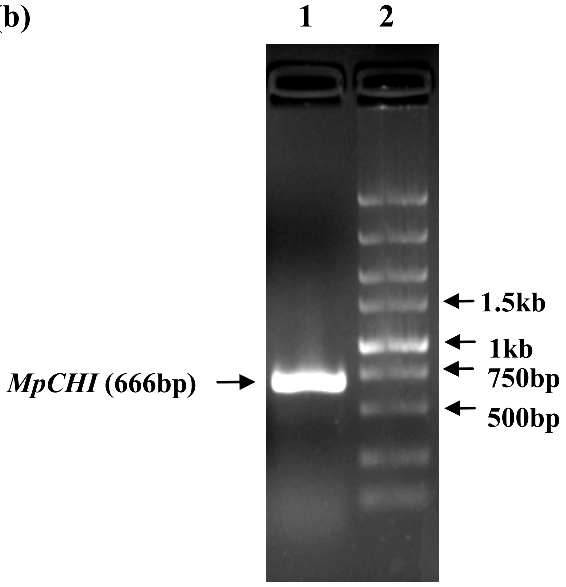

**Figure S2.** MpCHI could not improve the salt tolerance of yeast Δhog1 mutant. Picture was photographed 2 days after NaCl treatment. Up wild-type (WT) yeast transformants were set as control. WT + pYES2: empty vector transformed WT yeast control; WT + pYES2-MpCHI: pYES2-MpCHI transformed WT yeast; Δhog1 + pYES2: empty vector transformed Δhog1 control; Δhog1 + pYES2-MpCHI: pYES2-MpCHI transformed Δhog1 yeast.

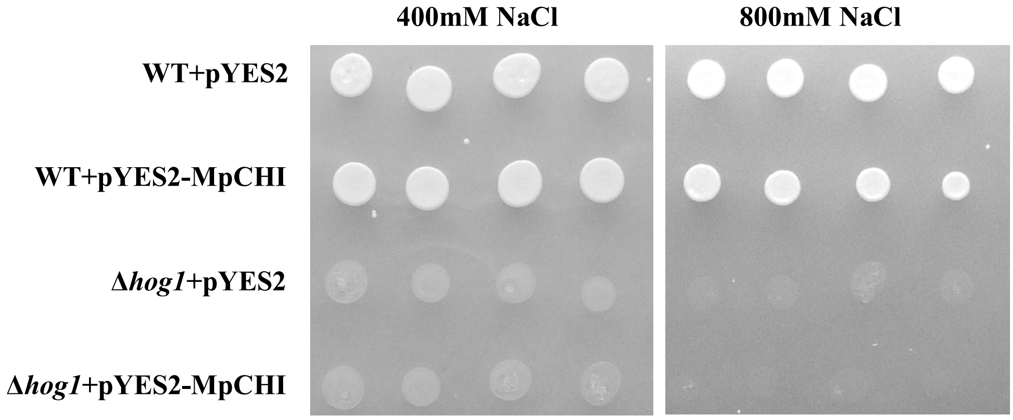


**Figure 3.** The MpCHI mRNA expression in yeast transformants were confirmed by RT-PCR. Bottom, yeast ACT1. Lane 1, blank control; Lane 2–5, yeast cultured in SC medium with glucose as carbon source; Lane 6–9, yeast cultured in SC medium with galactose as carbon source; Lane 10–13, yeast cultured in SC medium with galactose as carbon source and supplied with 500 mM NaCl; Lane 2, 6 and 10 are pYES2-vector transformed Δnha1; Lane 3, 7 and 11 are pYES2-MpCHI transformed Δnha1; Lane 4, 8 and 12 are pYES2-vector transformed Δnhx1; Lane 5, 9 and 13 are pYES2-MpCHI transformed Δnhx1.

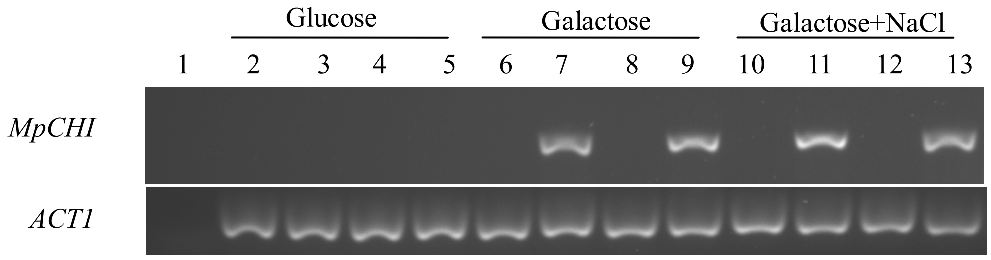


© 2013 by the authors; licensee MDPI, Basel, Switzerland. This article is an open access article distributed under the terms and conditions of the Creative Commons Attribution license (http://creativecommons.org/licenses/by/3.0/).
